# Comparative Study of the Effect of Sample Pretreatment and Extraction on the Determination of Flavonoids from Lemon (*Citrus limon*)

**DOI:** 10.1371/journal.pone.0148056

**Published:** 2016-01-25

**Authors:** Carlos A. Ledesma-Escobar, Feliciano Priego-Capote, María D. Luque de Castro

**Affiliations:** 1 Department of Analytical Chemistry, Annex C-3, Campus of Rabanales, University of Córdoba, Córdoba, Spain; 2 University of Córdoba Agroalimentary Excellence Campus, ceiA3, Campus of Rabanales, E-14071, Córdoba, Spain; 3 Institute of Biomedical Research Maimónides (IMIBIC), Reina Sofía Hospital, University of Córdoba, E-14071, Córdoba, Spain; The University of Tokyo, JAPAN

## Abstract

**Background:**

Flavonoids have shown to exert multiple beneficial effects on human health, being also appreciated by both food and pharmaceutical industries. Citrus fruits are a key source of flavonoids, thus promoting studies to obtain them. Characteristics of these studies are the discrepancies among sample pretreatments and among extraction methods, and also the scant number of comparative studies developed so far.

**Objective:**

Evaluate the effect of both the sample pretreatment and the extraction method on the profile of flavonoids isolated from lemon.

**Results:**

Extracts from fresh, lyophilized and air-dried samples obtained by shaking extraction (SE), ultrasound-assisted extraction (USAE), microwave-assisted extraction (MAE) and superheated liquid extraction (SHLE) were analyzed by LC–QTOF MS/MS, and 32 flavonoids were tentatively identified using MS/MS information. ANOVA applied to the data from fresh and dehydrated samples and from extraction by the different methods revealed that 26 and 32 flavonoids, respectively, were significant (*p*≤0.01). The pairwise comparison (Tukey HSD; *p*≤0.01) showed that lyophilized samples are more different from fresh samples than from air-dried samples; also, principal component analysis (PCA) showed a clear discrimination among sample pretreatment strategies and suggested that such differences are mainly created by the abundance of major flavonoids. On the other hand, pairwise comparison of extraction methods revealed that USAE and MAE provided quite similar extracts, being SHLE extracts different from the other two. In this case, PCA showed a clear discrimination among extraction methods, and their position in the scores plot suggests a lower abundance of flavonoids in the extracts from SHLE. In the two PCA the loadings plots revealed a trend to forming groups according to flavonoid aglycones.

**Conclusions:**

The present study shows clear discrimination caused by both sample pretreatments and extraction methods. Under the studied conditions, liophilization provides extracts with higher amounts of flavonoids, and USAE is the best method for isolation of these compounds, followed by MAE and SE. On the contrary, the SHLE method was the less favorable to extract flavonoids from citrus owing to degradation.

## Introduction

Flavonoids are compounds widely distributed in plants, giving place to beverages obtained from them (like wine, juice or beer) rich in these compounds. They are pigments responsible for the color and flavor of flowers and fruits [1]. Reports on the presence of flavonoids in citrus date since the forties of the past century [2], and their antioxidant properties, mainly as protectors of ascorbic acid in juices, are known and discussed since the sixties [3]. Nevertheless, it was not until the nineties when flavonoids started to reach importance thanks to the multiple beneficial effects on human health associated to their consumption. The numerous studies dealing with the bioactive properties of flavonoids have been mainly associated to the reduction of the risk of different types of cancer and cardiovascular diseases [4], and to their antioxidant, anti-inflammatory and radical-scavenging activity [5]. The latest investigations on the beneficial effects of flavonoids pointed out the actions of some of them, like quercetin, in anti-diabetic and anti-obesity treatments [6, 7], and as protectors of gastric epithelial cells [8]. In addition, flavonoids have shown anti-microbial effects which, linked to their antioxidant capacity, make them attractive for the food industry as natural preservatives or additives in functional foods [9].

Depending on their chemical structure, flavonoids are classified into six subclasses: flavonones, flavones, flavonols, flavans, isoflavones and anthocyanidins. Citrus fruits are rich in flavonoids [10] mainly belonging to the first three subclasses [11]. Taking into account that citrus cultivation is extended around the world, with an annual production of approximately 115 million tons [12], this fruit is a key source of these compounds. This is the reason why a large number of studies on extraction of citrus flavonoids, with or without assistance of any type of energy, has been carried out in the last two decades, mainly devoted to either establishing the total phenolic content (therefore, flavonoids being only a part of the pursued aim) or to the determination of a small number of flavonoids mainly based on liquid chromatography separation and molecular absorption detection [13]. Such is the case of the research by Ye et al. in 2011 [14] on identification of bioactive compounds from mandarins (*viz*. six flavonoids and seven phenolic acids); or that developed by Sdiri et al. in 2012 on the phenolic composition of mandarins restricted to thirteen flavonoids [15]. A more representative study on the composition of flavonoids in citrus is that reported by Abad-García et al. in 2012, who identified 45 flavonoids distributed among four varieties of fruits [16].

The extraction of citrus components strongly depends on the previous sample treatment, aspect that has been scantly taken into account in previous studies. Despite the large number of publications dealing with extraction of citrus flavonoids, only in few of them different sample pretreatment methods were compared. Most of the studies, focused on the effect of different air-drying temperatures for sample dehydration on the abundances of flavonoids, suggested that high temperatures promote the cleavage of the glycosidic bond and release of the aglycone form [17, 18]. On the other hand, studies on extraction methods have been characterized either by using an overall method to quantify the extracted compounds or by monitoring a few individual flavonoids. Example of the former is the study by Dahmoune et al. in 2013, who optimized microwave-assisted extraction (MAE) and ultrasound-assisted extraction (USAE) methods and compared the extracts thus obtained with those provided by conventional shaking extraction (SE); but always by monitoring the total phenolic content by the Folin–Ciocalteu method [19]. Similarly, García-Castello et al. in 2015, compared USAE and SE for both total phenolic content and total antioxidant activity and also quantified six flavonoids which were not used for comparisons [20].

In an attempt to clarify the effect of sample pretreatment (lyophilization, air-drying or blend) and auxiliary energies (ultrasound, microwaves or temperature+pressure) on the extraction of flavonoids from lemon (*Citrus limon*), the present research was aimed at establishing similarities/dissimilarities among the proposed methods by multivariate analysis, based on the profile of thirty two flavonoids tentatively identified in all the extracts from citrus.

## Materials and Methods

### Samples

Edible lemons (*Citrus limon*) were purchased in a local market in Córdoba, Spain (January, 2014). Specifications of the product: place of cultivation, Murcia, Spain; size, 53–67 mm; preservative, imazalil. The fruits were washed, cut in slices, and either lyophilized or air-dried (45°C) to constant weight and finally grinded (particle diameter ≤ 0.5 mm). The powder was stored in the dark at –20°C until use. Also, fresh lemons were ground in a blender to obtain a homogenous mixture and used immediately in all cases.

### Reagents

All solvents were LC grade or higher when required. Ethanol and formic acid were from Scharlab (Barcelona, Spain); acetonitrile (ACN) and methanol from Fluka (Buches, Switzerland). Deionized water (18 MΩ•cm) from a Millipore Milli-Q water purification system (Bedford, MA, USA) was used to prepare the mobile chromatographic phases and extractant mixtures.

### Apparatus and Instruments

The reference extracts were obtained by shaking using a Vibromatic reciprocating shaker (Selecta, Barcelona, Spain). Ultrasound was applied by a Branson 450 digital sonifier (20 kHz, 450 W) equipped with a cylindrical titanium-alloy probe (12.70 mm diameter). Microwave assistance was provided by a focused microwave digester (200 W) Microdigest 301 (Prolabo, Paris, France). Superheated liquid extractions were carried out by a laboratory-made dynamic extractor consisting of the following units: (a) an extractant supplier, (b) a high pressure pump (Shimadzu LD-AC10), which propels the extractant through the system, (c) a switching valve placed next to the pump to develop static extractions if required, (d) a stainless-steel cylindrical extraction chamber (550 × 10 mm inner diameter and 4.3 mL internal volume), where the sample is placed (this chamber is closed at both ends with screws whose caps contain cotton-made filters to ensure the sample is not carried away by the extractant), (e) a restriction valve to maintain the desired pressure in the system, (f) a cooler made of a stainless-steel tube (1 m length and 0.4 mm inner diameter) and refrigerated with water, and (g) a gas chromatograph oven (Konix, Cromatix KNK-2000), where the extraction chamber is placed and heated.

The analytical equipment consisted of an Agilent 1200 series LC coupled to an electrospray ionization source and a quadrupole–time of flight detector 6540 Agilent Q–TOF (LC–QTOF MS/MS).

### Extraction

Lemon samples (1 g dry weight each) were extracted in 20 mL of solvent. Taking into account that flavonoids are polar/midpolar compounds, mixtures of ethanol and water are commonly used as extractants in order to obtain extracts useful for being used as food supplements [21]. The suited conditions for extraction of flavonoids by USAE (extraction time of 5 min; 60% ethanol in water; amplitude 70% and duty cycle of 0.8 s s^–1^); MAE (6 extraction cycles; 68% ethanol in water and 170 W); SHLE (15 min; 73% ethanol in water and 150°C) and shaking extraction (SE) (60 min: 60% ethanol in water), previously determined by the authors using a desirability study to maximize the concentration of five flavonoids abundant in lemon [22], were those used in the present study, which also used conventional SE to evaluate the effect of sample pretreatment on the flavonoid composition. The effect of USAE, MAE and SHLE on the extraction of flavonoids was compared using lyophilized lemon samples, for which the extracts, obtained in duplicate under the suited working conditions for each method, were used to obtain the flavonoids profile as provided by LC–QTOF MS/MS analysis.

### LC–QTOF MS/MS Analysis

Chromatographic separation was performed by using an Inertsil ODS-2 C18 analytical column (250 × 4.6 mm i.d. 5 μm particle) from Análisis Vínicos (Tomelloso, Ciudad Real, Spain). The injection volume was 10 μL, and the mobile phase was deionized water (A) and ACN (B), both with 0.1% of formic acid as ionization agent, at a constant flow rate of 1 mL min^–1^. The gradient was as follows: 4% to 10% B in 5 min; change from 10% to 25% B in 30 min; from 25% to 100% B in 15 min and constant 100% B for 5 min.

The dual ESI source operated in both positive and negative ionization modes under the following conditions: nebulizer gas at 40 psi, drying gas flow rate and temperature at 12 L min^–1^ and 325°C, respectively. The capillary voltage was set at 3500 V, while the fragmentor, skimmer, and octapole voltages were fixed at 130, 65, and 750 V, respectively. The data were acquired in centroid mode in the extended dynamic range (2 GHz). Full scan was carried out at 6 spectra s^–1^ within the *m/z* range of 40–1700, with subsequent activation of the three most intense precursor ions (allowed charge: single or double) by MS/MS using a collision energy of 20 eV and 40 eV at 3 spectra/s within the *m/z* range 30–1700. An active exclusion window was programmed after the first spectrum and released after 0.75 min to avoid repetitive fragmentation of the most intense precursor ions, thus increasing the detection coverage. To assure the desired mass accuracy of recorded ions, continuous internal calibration was performed during analyses with the use of signals at *m/z* 121.0509 (protonated purine) and *m/z* 922.0098 [protonated hexakis (1H, 1H, 3H-tetrafluoropropoxy)phosphazine or HP-921] in the positive ionization mode; and *m/z* 112.9856 (trifluoroacetic acid anion) and *m/z* 1033.9881 (HP-921) in the negative mode.

### Data Processing and Statistical Analysis

MassHunter Workstation software (version B6.00 Profinder, Agilent Technologies, Santa Clara, CA, USA) was used to process all the data obtained by LC–QTOF in auto MS/MS mode. Treatment of the raw data file started by extraction of potential molecular features (MFs) with the applicable algorithm included in the software. The recursive extraction algorithm considered all ions exceeding 5000 and 10000 counts as cut-off in both positive and negative modes, respectively. Additionally, the isotopic distribution to consider a MF as valid should be defined by two or more ions (with a peak spacing tolerance of 0.0025 *m/z*, plus 10.0 ppm in mass accuracy). Apart from [M+H]^+^ and [M−H]^−^ ions, adducts formation in the positive (Na^+^) and negative ionization (HCOO^−^, Cl^−^) modes, as well as neutral loss by dehydration were included to identify features corresponding to the same potential metabolite. Thus, ions with identical elution profiles and related *m/z* values (representing different adducts or isotopes of the same compound) were extracted as entities characterized by their retention time (RT), intensity in the apex of the chromatographic peaks and accurate mass. Background contribution was removed by subtraction of MFs linked to the blank. Then, the recursion step assured correct integration of the entities in all analyses. Raw data files, containing the area for each entity characterized by *m/z* and RT, were created in compound exchange format (.cef files) for each analysis and exported into the Mass Profiler Professional (MPP) software package (version 2.0, Agilent Technologies, Santa Clara, CA, USA) for further processing. Normalization by logarithmic transformation (log2) was used as pre-processing step. Statistical analysis included the ANOVA test applied to find the number of significant flavonoids (*p*≤0.01), and pairwise combinations (Tukey HSD) to identify equal concentration of flavonoids between extraction methods. Also, unsupervised analysis by Principal Component Analysis (PCA) was used to find out the main source of variability in the data set and detect clusters.

Once all MFs were extracted and aligned, the software MassHunter Qualitative was used for the targeted extraction of MS/MS information associated to them in the whole set of analyses. This information was used for the tentative identification of flavonoids by searching in the METLIN MS and MS/MS (http://metlin.scripps.edu), MassBank MS/MS (http://www.massbank.jp) and ReSpect MS/MS (http://spectra.psc.riken.jp) databases.

## Results and Discussion

To compare the effect of the two methods for sample dehydration (between them and with a blended fresh sample) and the three extraction methods assisted by the different energies, extracts obtained under the specified suited conditions were analyzed by LC–QTOF MS/M for identification of flavonoids. A list including 32 tentatively identified flavonoids and their identification parameters are shown in Table 1.

**Table 1:** Identification parameters—flavonoid name, neutral mass, retention time (RT), adduct formed, precursor ion (*m/z*) and main product ions (*m/z*)—, for the 32 flavonoids tentatively identified.

Compound name	Formula	Neutral mass	RT	Precursor Ion (*m/z*)	Adduct	Main *m/z* fragments		
**FLAVANONES**								
Eriocitrin	C27H32O15	596.1740	20.88	597.1811	**[M+H]+**	289.0699	153.0179	163.0390
Eriodictyol-Glu-Rha-Glu	C33H42O20	758.2265	21.47	757.2200	**[M-H]-**	287.0541	449.1067	595.1663
Neoeriocitrin	C27H32O15	596.1744	27.3	295.166	**[M-H]-**	287.0534	151.0012	135.0422
Eriodictyol-Neo-Rha	C33H42O19	742.232	27.49	743.2393	**[M+H]+**	435.1307	289.0716	195.0294
Naringin	C27H32O14	580.1782	32.48	579.1717	**[M-H]-**	271.0590	151.0003	119.0474
Hesperidin	C28H34O15	610.1884	33.42	609.1818	**[M-H]-**	301.0697	325.0687	343.0822
Hesperetin	C16H14O6	302.0801	34.76	303.0866	**[M+H]+**	153.0181	177.0541	117.0349
Hesperetin-7-O-Rha	C22H24O10	448.1368	34.85	449.1442	**[M+H]+**	303.0858	177.0545	153.0183
Neohesperidin	C28H34O15	610.1893	35.04	609.1827	**[M-H]-**	301.0703	325.0707	125.0183
**FLAVONES**								
Apigenin-Glu-Rha-Glu	C33H40O19	786.2218	22.77	785.2085	**[M+FA-H]-**	269.0424	577.1554	431.0949
Luteolin-Rut-Glu	C33H40O20	756.2105	23.01	755.2041	**[M-H]-**	285.0385	593.1509	
Homoorientin	C21H20O11	448.0992	25.34	447.0908	**[M-H]-**	357.0591	327.0478	297.0367
Orientin	C21H20O11	448.1006	25.77	447.0915	**[M-H]-**	357.0566	327.0487	298.0448
Vitexin -O-xyloside	C26H28O14	564.1471	28.15	563.1396	**[M-H]-**	293.0422	59.0121	311.0516
Vitexin	C21H20O10	432.1058	28.60	431.0952	**[M-H]-**	311.0536	283.0562	341.0636
Vitexin-2-Rha	C27H30O14	578.1627	28.91	577.1559	**[M-H]-**	293.0427	413.0883	59.0117
Luteolin-Glu-Rha	C27H30O15	594.1583	29.61	595.1659	**[M+H]+**	287.0552	449.1074	
Luteolin-Neo	C27H30O15	594.1583	29.85	593.1507	**[M-H]-**	285.0382	284.0293	151.0005
Diosmetin-Glu	C22H22O11	462.1146	31.20	497.0871	**[M+Cl]-**	461.1071	341.0631	298.0458
Rhoifolin	C27H30O14	578.1623	34.11	577.1555	**[M-H]-**	269.0435	311.0527	85.0270
Diosmin	C28H32O15	608.1729	34.90	607.1657	**[M-H]-**	299.0537	284.0282	301.0697
Neodiosmin	C28H32O15	608.1745	35.99	607.1663	**[M-H]-**	299.0527	284.0285	509.9860
Diosmetin-Glu-Rha	C28H32O15	608.1744	39.79	609.1819	**[M+H]+**	301.0706	463.1233	153.0182
**FLAVANOLS**								
Quercetin-Glu-Rha-Glu	C33H40O21	772.2055	17.26	771.1984	**[M-H]-**	609.1442	462.0776	341.0449
Rutin	C27H30O16	610.1528	29.70	609.1456	**[M-H]-**	300.0247	301.0330	271.0259
Limocitrin-Neo	C29H34O17	654.1790	33.36	653.1723	**[M-H]-**	345.0577	330.0332	301.0684
Spinacetin-Glu-HMG-Glu	C35H42O22	814.2166	33.52	813.2046	**[M-H]-**	651.1548	549.1258	345.0579
Limocitrin-Glu-HMG-Glu	C35H42O22	814.2167	33.98	813.2046	**[M-H]-**	651.1548	549.1258	345.0579
Isorhamnetin-3-O-Neo	C28H32O16	624.1678	34.32	623.1631	**[M-H]-**	315.0491	300.0238	271.0202
Limocitrol-Glu-HMG	C18H16O9	682.1744	37.60	681.1630	**[M-H]-**	375.0699	360.0472	537.1240
Limocitrin-HMG-Glu	C29H32O17	652.1640	37.80	651.1566	**[M-H]-**	345.0593	549.1233	507.1117
Quercetin-3-O-Neo	C27H30O16	610.1885	39.74	609.1844	**[M-H]-**	301.0691	151.0023	

Glu, glucoside; Neo, neohesperidoside; Rut, rutinoside; HMG, 3-hydroxy-3-methyl-glutaryl.

### Effect of the Sample Pretreatment Procedures on the Extraction of Flavonoids

The effect of sample pretreatment on the profile of flavonoids was evaluated by ANOVA, which showed that the concentration of 26 out of the 32 tentatively identified flavonoids was significant *(p*≤0.01), as did the pairwise means comparison (Tukey HSD; *p*≤0.01), used to show the similarity of abundance between extracts. In short, this study revealed that the extracts obtained from air-drying samples contained 18 flavonoids with concentration different to both lyophilized and fresh samples. On the other hand, the comparison between extracts from lyophilized and fresh samples showed 24 flavonoids with significantly different concentration. Neodiosmin and neohesperidin were more concentrated in the extracts from lyophilized samples, followed by fresh samples and being less abundant in the extracts from air-dried samples. On the contrary, limocitrin-HMG-Glu and limocitrol-Glu-HMG were more concentrated in the extracts from air-drying samples, followed by fresh samples. The complete dataset used to evaluate the effect of sample pretreatment on the extraction of flavonoids are shown in S1 Table.

**Fig 1:**
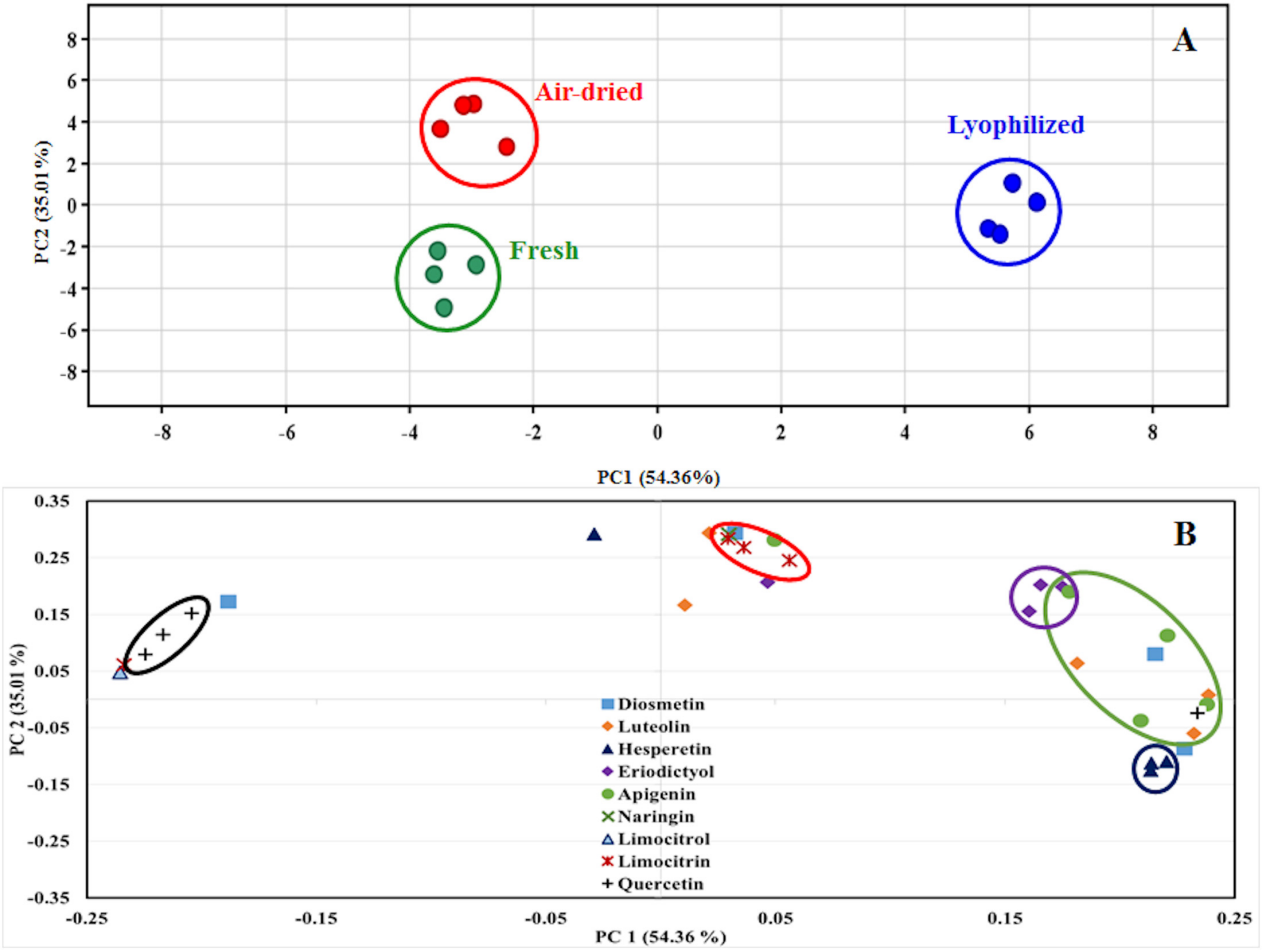
Scores (A) and loadings (B) PCA plots comparing the different treatments prior to flavonoids extraction.

To clarify the effect of sample pretreatment on flavonoids extraction, an unsupervised analysis by PCA was applied to compare the flavonoid profiles in the extracts provided by samples differently pretreated. The scores plot (Fig 1A) shows a clear discrimination between the obtained profiles; the extracts from lyophilized samples are clearly discriminated from the rest along component 1 (PC1), while extracts from both fresh and air-drying samples are discriminated along component 2 (PC2). The proximity in the PCA between the scores provided by the methods reveals the similarity between them. It can be seen that despite the ANOVA test reveals the same number of flavonoids with different concentration in the comparison between air-drying and either lyophilized or fresh samples, the PCA indicates that extracts from air-drying samples are more similar to those from fresh samples than those from lyophilized samples. The PCA explains 89.37% (PC1 = 54.36% and PC2 = 35.01%) of the total variability in the 2D-plot. Although the results from PCA are often difficult to interpret, it is possible to obtain reasonable conclusions from them in addition to clusters formation. As can be seen in the loadings plot (Fig 1B), the most influential flavonoids in the ANOVA test are in opposite sides on the PC1: neodiosmin and neohesperidin, the most abundant in lyophilized samples, are in the positive side (quadrant four); while limocitrin-HMG-Glu and limocitrol-Glu-HMG, the most abundant in both fresh and air-drying samples, are in the negative side (quadrant two). These four flavonoids are the main responsible for discrimination between extracts from lyophilized samples and the other two. The differences can be explained by the flavonoids pathway and the intrinsic characteristics of the extraction methods. Regarding lemons, flavanones are the first in the biosynthesis of flavonoids, followed by flavones and then flavonols. Prior to lyophilization the sample was frozen at –80°C, thus promoting a significant reduction of lemon metabolism; on the contrary, by air-drying the sample was heated at 45°C, which accelerates enzymatic reactions, thus favoring flavanols production.

On the other hand, discrimination between extracts from fresh and air-drying samples, occurring in PC2, could be achieved by the abundance of hesperidin, naringin, luteolin-rutinoside and limocitrin derivatives, which are the most influential flavonoids on this component. Also, an apparent trend to grouping by aglycones exists, more evident in derivatives from hesperetin, limocitrin, apigenin, eriodictyol and quercetin. The differences in individual flavonoids caused by the sample pretreatments under study are discussed below.

### Differences in the Abundance of Flavanones in Extracts from Differently Pretreated Samples

**Fig 2:**
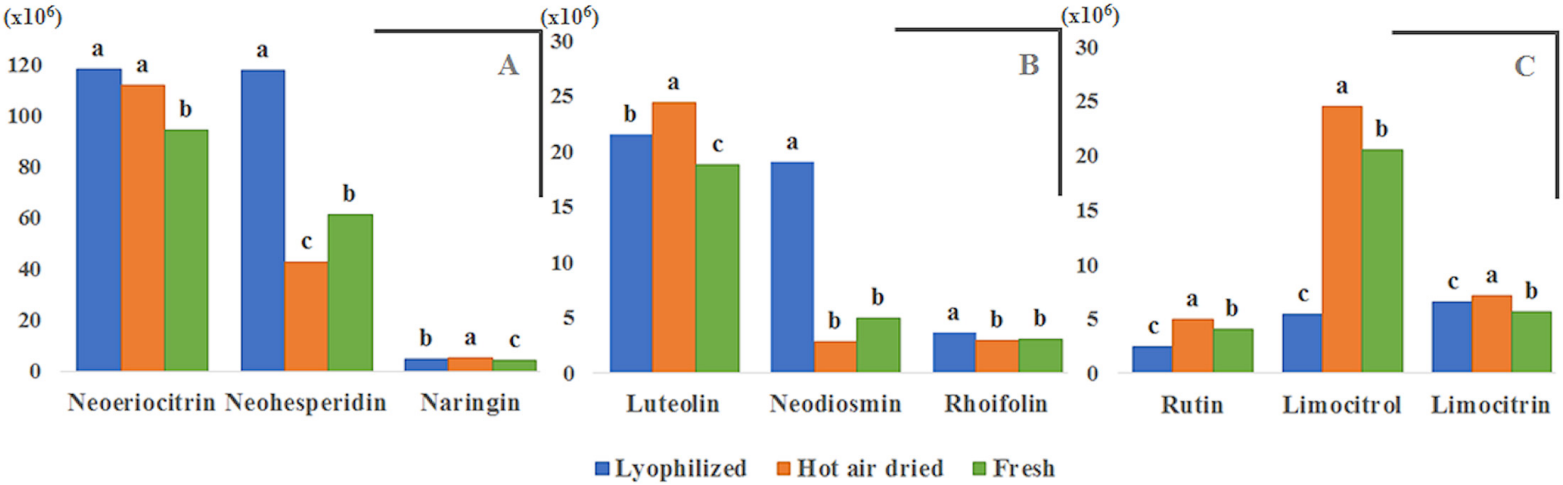
Comparison of the most abundant (A) flavanones, (B) flavones and (C) flavanols, from lemon extracted by SE after different pretreatments. Bars with different letter for the same flavonoid are significantly different (Tukey HSD, *p*≤0.05).° neohesperidoside; * glucoside–3-hydroxy-3-methyl-glutaryl.

This subclass of flavonoids is the most abundant in citrus fruits and includes some of the most characteristic flavonoids in lemon as naringenin or hesperetin derivatives [23]. After tentative identification of naringin, four derivatives from eriodictyol and four derivatives from hesperetin, the most abundant of all these flavonoids showed to be neohesperidin and neoeriocitrin. The comparison of means (Tukey HSD; *p*<0.05) revealed that the abundance of neohesperidin is significantly different for all sample pretreatments being the extracts from lyophilized samples the richest in this flavonoid, followed by fresh samples. The behavior of neoeriocitrin is divided into two homogeneous groups: group *a* encompasses extracts obtained from dehydrated samples, which show an abundance greater than extracts from fresh samples (group *b*). The concentration of naringin depended on the sample pretreatment, being the richest in this flavonone the extracts from air-dried samples and the poorest those from fresh samples. For this subclass, the extracts from lyophilized samples were those with the highest abundance in all flavonones; on the contrary, the extracts from fresh samples were those with the lowest abundance, as shown in Fig 2A.

### Differences in the Abundance of Flavones in Extracts from Differently Pretreated Samples

This subclass of flavonoids is synthesized from flavanones as the direct biosynthetical precursor by the abstraction of two hydrogen atoms, mainly catalyzed by flavone synthase [24]. In this study, the tentative identification indicates the presence of four diosmetin glucosides; five luteolin glucosides and five apigenin glucosides including vitexin xyloside as the only one identified flavonoid linked to pentose.

According to the metabolic pathway of flavonoids, flavone–neohesperidosides can be synthesized from their corresponding flavonone–neohesperidosides, as reported by Martens et al. in 2005 [24]; naringenin can be converted into apigenin by dehydrogenation catalyzed by flavone synthase. Similarly, neohesperidin (hesperetin 7-O-neohesperidoside) can be converted into neodiosmin (diosmetin 7-O-neohesperidoside). Luteolin-neohesperidoside was the most abundant flavone in this study, the biosynthetic pathway being dehydrogenation of eriodictyol catalyzed by a flavone synthase or hydroxylation of apigenin, catalyzed by a flavonoid hydroxylase [25]. The pairwise comparison showed that the average abundance for all treatments is significantly different among them, the extracts from air-dried and fresh samples showed the greater and lower abundance, respectively, of luteolin-neohesoeridoside. Neodiosmin was the second most abundant flavone identified in this study, which was more abundant in extracts from lyophilized samples (group *a*), and with no significant differences between air-dried and fresh samples. Taking into account that neohesperidin is both the most abundant flavanone in lemon and the precursor of neodiosmin, it seems to be one of the most abundant flavone in lemon and the extracts from lyophilized samples were the most concentrated in it. Regarding to apigenin glucosides, rhoifolin (apigenin-neohesperidoside) was the most abundant and showed the same extraction behavior as neodiosmin. Rhoifolin could be synthesized from naringin by a flavone synthase; then being hydroxylated to produce luteolin-neohesperioside [25]. The abundance of the three main flavones is shown in Fig 2B.

### Differences in the Abundance of Flavanols in Extracts from Differently Pretreated Samples

According to the general flavonoids pathway, flavonols are synthesized from flavanones by a two-step synthesis which starts with hydroxylation caused by a flavonone hydroxylase, followed by dehydrogenation catalyzed by a flavonol synthase [24]. In this study, three quercetin and limocitrin glucosides were tentatively identified as the main flavonols in lemon; also glucosides from limocitrol, isorhamentin and spinacetin (the latter being an isomer of limocitrin) were tentatively identified. Limocitrin and limocitrol (both conjugated to a glucoside and a 3-hydroxy-3-methyl-glutaryl) and rutin were the most abundant flavonols identified in this study. They were more abundant in extracts from air-dried samples, and less abundant in lyophilized samples. In general, this behavior was the same for all flavonols, except for quercetin-nesperidoside, which was more abundant in extracts from lyophilized samples. The abundance of the three main flavonols is shown in Fig 2C, while the complete set of results from the ANOVA test for all individual flavonoids and their pairwise comparison are in Table 2.

**Table 2:** Average of abundance and pairwise comparison (Tukey HSD, *p*≤0.05), of the flavonoids identified in the extracts from different sample pretreatments of the sample. Values with different letter for the same flavonoid (same row) are significantly different (Tukey HSD, *p*≤0.05). Values are means±SD×10^6^ (*n* = 4).

Compound	Lyophilized	Air-dried	Fresh
**FLAVANONES**			
***Neoeriocitrin***	117.98±3.14a	111.48±2.2a	94.36±6.15b
***Neohesperidin***	117.33±5.48a	42.53±1.19c	61.22±1.59b
***Eriodictyol-Glu-Rha-Glu***	6.19±0.38a	5.55±0.14a	4.27±0.28b
***Naringin***	4.51±0.22b	4.97±0.16a	3.98±0.12c
***Hesperetin***	3.64±0.15a	1.38±0.15c	2.04±0.08b
***Hesperetin-7-O-Rha***	7.03±0.88a	2.49±0.34b	3.64±0.25b
***Eriocitrin***	0.64±0.05a, b	0.74±0.12a	0.5±0.12b
***Hesperidin***	1.41±0.05b	1.65±0.09a	1.31±0.04b
***Eriodictyol-Neo-Rha***	0.43±0.03a	0.39±0.02a, b	0.33±0.04b
**FLAVONES**			
***Luteolin-Neo***	21.49±0.72b	24.37±1.59a	18.7±0.99c
***Diosmetin-Glu***	7.67±0.34b	8.62±0.48a	6.52±0.29c
***Luteolin-Glu-Rha***	21.2±1.33a	8.22±0.53b	9.89±0.24b
***Rhoifolin***	3.64±0.26a	2.87±0.14b	3.04±0.06b
***Neodiosmin***	24±2.64a	2.78±0.12b	4.92±0.1b
***Vitexin -O-xyloside***	2.98±0.07a	2.65±0.15b	2.11±0.19c
***Diosmin***	0.73±0.02c	1.25±0.07a	0.94±0.08b
***Vitexin***	1.23±0.09b	1.43±0.06c	0.9±0.05a
***Apigenin-Glu-Rha-Glu***	1.58±0.08a	0.76±0.02b	0.78±0.08b
***Diosmetin-Glu-Rha***	0.9±0.02a	0.55±0.05b	0.37±0.02c
***Luteolin-Rut-Glu***	1.25±0.09a	0.52±0.01b	0.49±0.01b
***Vitexin-2-Rha***	0.72±0.04a	0.5±0.03b	0.4±0.04c
***Homoorientin***	1.21±0.09a	1.02±0.06a, b	0.97±0.12b
***Orientin***	0.7±0.09a	0.77±0.15a	0.63±0.07a
**FLAVANOLS**			
***Quercetin-Glu-Rha-Glu***	0.55±0.03b	1.03±0.1a	0.91±0.02a
***Rutin***	2.45±0.07c	4.99±0.28a	3.99±0.28b
***Limocitrin-Neo***	1.29±0.06a	1.44±0.11a	1.09±0.02b
***Spinacetin-Glu-HMG-Glu***	6.5±0.21a, b	6.85±0.61a	5.64±0.23b
***Limocitrin-Glu-HMG-Glu***	6.51±0.25a, b	7.13±0.63a	5.6±0.18b
***Isorhamnetin-3-O-Neo***	1.31±0.04c	2.36±0.17a	1.83±0.07b
***Limocitrol-Glu-HMG***	5.39±0.15c	24.51±2.01a	20.49±1.85b
***Limocitrin-HMG-Glu***	4.29±0.11c	16.92±1.36a	13.47±1.32b
***Quercetin-3-O-Neo***	0.48±0.02a	0.24±0.03b	0.24±0.01b

Glu, glucoside; Neo, neohesperidoside; Rut, rutinoside; HMG, 3-hydroxy-3-methyl-glutaryl.

In general, these results suggest that sample dehydration provide extracts with higher amounts of flavonoids than fresh samples, behavior attributable to the reduction of water activity in dehydrated samples, which decrease the activity of polyphenol oxidase [26]. Also, despite of flavonoids are thermolabile compounds, air-dried samples provide extracts with lower amounts of flavonoids than lyophilized samples.

### Effect of the Different Extraction Methods on Flavonoids Removal

The results discussed above showed that lyophilized samples provide extracts with higher amounts of flavonoids than either air-dried or fresh samples; thus leading to decide the use of lyophilized samples for evaluation of the effect of USAE, MAE and SHLE on the removal of these compounds.

The extracts obtained with the help of the different energies were analyzed by LC–QTOF MS/MS and compared with the results from the reference extract obtained by SE. Based on the MS/MS information, the tentative identification of 32 flavonoids, which constituted the data set used for the corresponding statistical analysis, was carried out. The complete dataset used to evaluate the effect of the different auxiliary energies on the extraction of flavonoids are shown in S2 Table.

The effect of auxiliary energies on the profile of flavonoids was evaluated by ANOVA, which showed that all flavonoids in the data set were significant (*p*≤0.01), and the pairwise analysis revealed the similarity between extracts. The comparison of means (Tukey HSD; *p*<0.01) reveals that the extracts provided by USAE and MAE were the most similar to each other, since only 7 out of 32 entities were significantly different in this pair. Discrimination between the extraction methods was caused mainly by flavones, since 5 out of 7 significantly different flavonoids in this pair corresponded to this subclass; the other two were flavonoid derivatives from eriodictyol and diosmin. The extracts obtained by MAE and SE showed eleven entities significantly different between them. Once again the most different entities were within the flavones subclass, but in this case, three derivatives from hesperetin, two from diosmin and one from quercetin were also different. The comparison of USAE–SE showed 17 different entities, 8 of them belonging to flavones, 8 to flavanones, and only quercetin-3-O-neohesperidoside was from the flavanol subclass. In short, the method based on SHLE provided an extract with clear differences in the flavonoids profile as compared to the rest: 23 flavonoids significantly different from SE, 30 from USAE and 31 from MAE extracts.

**Fig 3:**
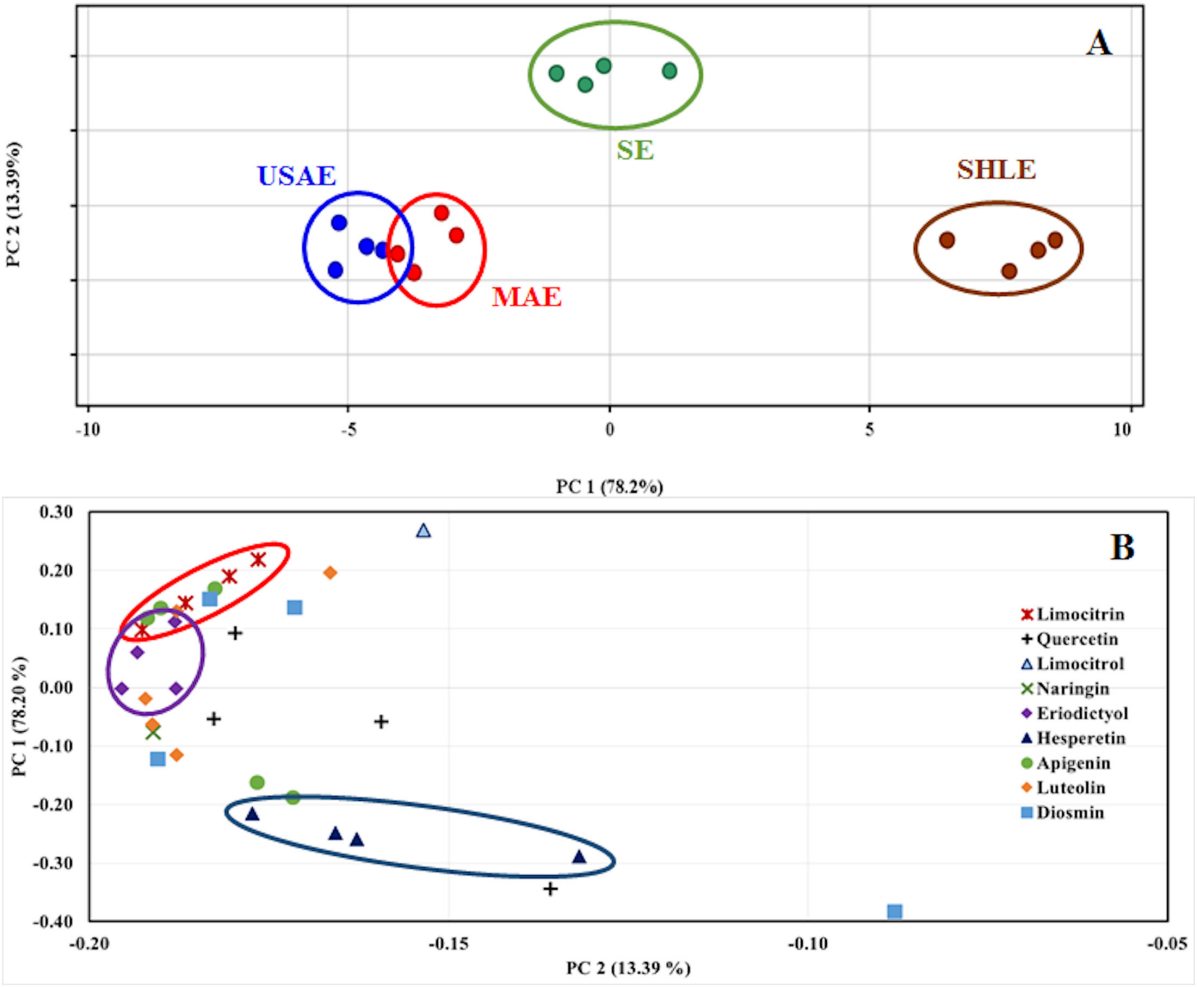
Scores (A) and loadings (B) PCA plots comparing the different methods for extraction of flavonoids.

For a better understanding of the effect of auxiliary energies on the extraction of flavonoids, a discrimination test was developed. With this purpose, an unsupervised analysis by PCA was applied to compare the flavonoid profiles provided by the different extraction methods. The scores plot (Fig 3A) shows a clear discrimination among all extraction methods. In agreement with the ANOVA test, the PCA reveals that USAE and MAE scores are closer to each other than the rest of scores; thus indicating that USAE and MAE were the most similar extracts; the cluster formed by the SHLE extracts is the furthest from the rest and the scores provided by SE extracts remain almost in between USAE–MAE and SHLE scores. The PCA explains 91.59% (PC1 = 78.20% and PC2 = 13.39%) of the total variability in the 2D-plot. As can been in the loadings plot (Fig 3B), all flavonoids are in the negative side of PC1, distributed across quadrants 2 and 3. This behavior suggests a lower abundance of flavonoids in the extracts from SHLE. Also, an apparent trend to grouping by aglycones exists, more evident in derivatives from hesperetin, limocitrin and eriodictyol. The differences in individual flavonoids obtained by the methods under study are discussed below.

### Differences in the Abundance of Representative Flavonoids in Lemon Extracts Obtained with the Help of Different Energies

**Fig 4:**
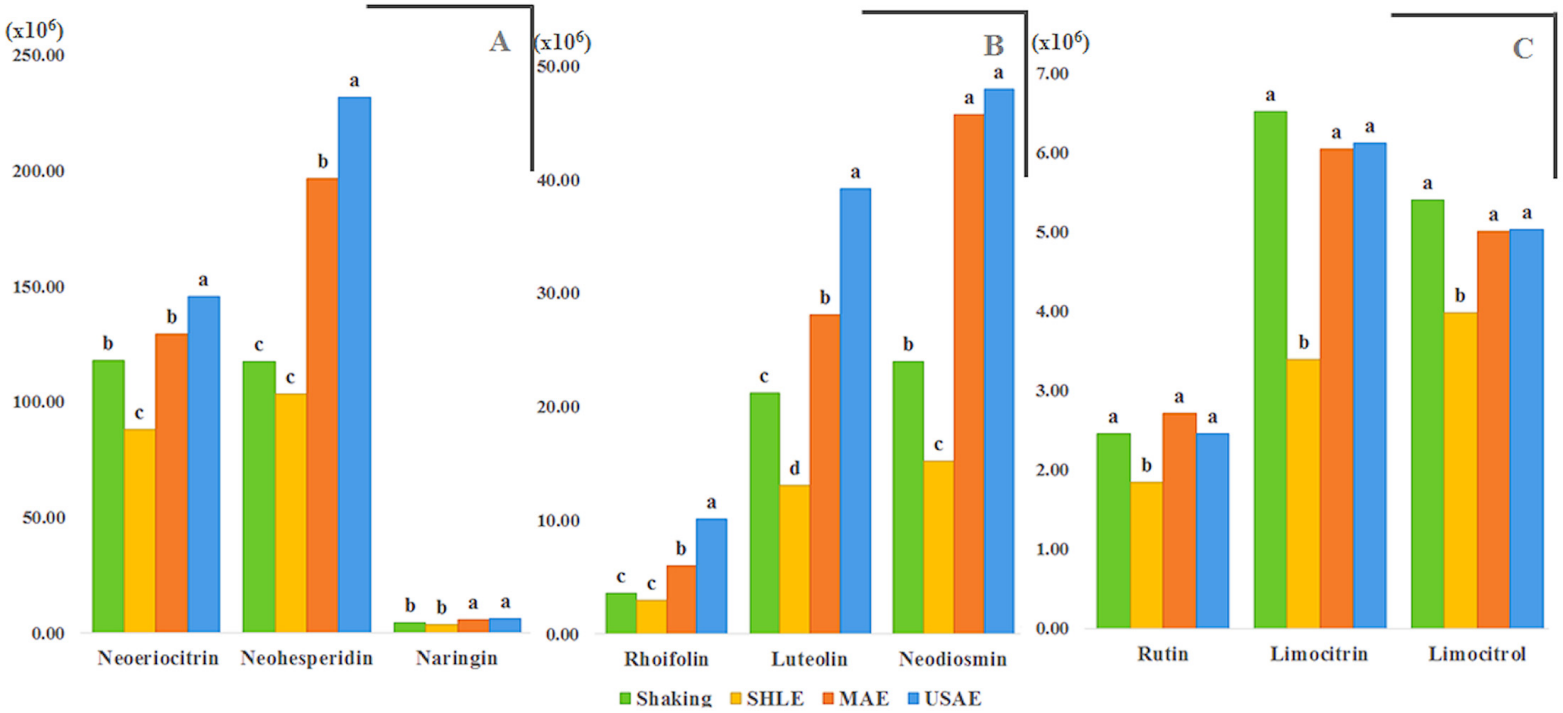
Comparison of the most abundant (A) flavanones, (B) flavones and (C) flavanols, obtained by extraction with different auxiliary energies at suited conditions. Bars with different letter for the same flavonoid are significantly different (Tukey HSD, *p*≤0.05).° neohesperidoside; * glucoside–3-hydroxy-3-methyl-glutaryl.

The effect of the extraction method on the individual flavonoids was analyzed by pairwise comparison of means (Tukey HSD; *p*<0.05). The results indicate that most of the flavonoids found in the study are better extracted by USAE, followed by MAE; SHLE was the less favorable to extract flavonoids from lemon. As examples of this behavior, the most abundant flavonoids, neohesperidin and neoeriocitrin, are divided into three homogeneous groups: group *a*, corresponding to the best extraction method, is represented for USAE extracts for both flavonoids; group *b* encompasses MAE extracts for neohesperidin and extracts obtained by MAE and SE for neoeriocitrin. The same trend was observed for luteolin-neohesperidoside and rhoifolin. Other flavonoids, like neodiosmin or naringin, are significantly equal extracted by MAE and USAE (group *a*), and limocitrin, limocitrol and rutin were not different in USAE, MAE and SE extracts (group *a*). In all mentioned cases, SHLE extracts represent the less desirable option (Fig 4). In general, 30 out of the 32 flavonoids identified in this study were more concentrated in USAE extracts. Among these 30 flavonoids, 11 of them were more concentrated only in USAE extracts; while in MAE extracts 19 of the 32 flavonoids identified had a concentration significant equal to that in USAE extracts, and two (homoorientin and dioemetin-glucoside-rhamnoside) were more concentrated than in USAE extracts. For the rest 19 flavonoids USAE and MAE extracts were significantly similar. On the contrary, SHLE extracts only provided concentration similar to USAE or MAE extracts for hesperidin, being lower the concentration for the other flavonoids. The complete set of results from the ANOVA test of all individual flavonoids and their pairwise comparison are in Table 3.

**Table 3:** Average of abundance and pairwise comparison (Tukey HSD, *p*≤0.05) of the flavonoids identified in the extracts obtained with the help of different energies. Values with different letter for the same flavonoid (same row) are significantly different (Tukey HSD, *p*≤0.05). Values are means×10^6^ ± RSD (%); (*n* = 4).

Compound	SE	SHLE	MAE	USAE
**FLAVANONES**
***Eriocitrin***	0.64±0.05c	0.33±0.02d	0.73±0.03b	0.84±0.02a
***Eriodictyol-Glu-Rha-Glu***	6.19±0.38b	2.81±0.26c	6.07±0.55b	7.88±0.38a
***Eriodictyol-Neo-Rha***	0.43±0.03b	0.26±0.03c	0.52±0.02a	0.6±0.04a
***Neoeriocitrin***	117.98±3.14b	87.76±2.61c	129.27±7.5b	145.62±5.11a
***Naringin***	4.51±0.22b	3.37±0.19b	5.95±0.9a	6.22±0.53a
***Hesperidin***	1.41±0.05b	1.49±0.08a, b	1.86±0.29a	1.86±0.06a
***Hesperetin***	3.64±0.15b	3.46±0.13b	7.54±0.34a	7.12±0.12a
***Hesperetin-7-O-Rha***	7.03±0.88b	6.89±0.75b	15.93±0.5a	14.85±0.63a
***Neohesperidin***	117.33±5.48c	103.4±5.76c	196.43±12.01b	231.62±10.49a
**FLAVONES**
***Apigenin-Glu-Rha-Glu***	1.58±0.08c	1.29±0.13c	2.2±0.17b	3.09±0.22a
***Luteolin-Rut-Glu***	1.25±0.09c	0.66±0.02d	1.53±0.08b	2.26±0.17a
***Homoorientin***	1.21±0.09a, b	0.67±0.03c	1.25±0.1a	1.06±0.05b
***Orienti***	0.7±0.09a	0.31±0.02b	0.76±0.05a	0.73±0.04a
***Vitexin -O-xyloside***	2.98±0.07a	1.24±0.06b	2.94±0.31a	3.08±0.17a
***Vitexin***	1.23±0.09a	0.53±0.06b	1.29±0.12a	1.33±0.07a
***Vitexin-2-Rha***	0.72±0.04b	0.32±0.02c	0.75±0.03b	0.86±0.04a
***Luteolin-Glu-Rha***	2.88±0.15c	1.88±0.06d	4.47±0.27b	6.45±0.23c
***Luteolin-Neo***	21.2±1.33c	13.06±0.26d	28.07±1.33b	39.17±0.45a
***Diosmetin-Glu***	5.35±0.19a	3.02±0.04b	5.76±0.4a	5.22±0.43a
***Rhoifolin***	3.64±0.26c	2.95±0.3c	6.03±0.37b	10.14±0.84a
***Diosmin***	0.73±0.02a	0.48±0.03b	0.7±0.08a	0.81±0.08a
***Neodiosmin***	24±2.64b	15.23±1.01c	45.69±1.84a	47.91±1.44a
***Diosmetin-Glu-Rha***	0.9±0.02d	2.38±0.3c	8.94±0.45a	4.07±0.3b
**FLAVANOLS**
***Quercetin-Glu-Rha-Glu***	0.55±0.03a, b	0.41±0.04b	0.71±0.13a	0.67±0.06a
***Rutin***	2.45±0.07a	1.83±0.11b	2.71±0.19a	2.45±0.08a
***Limocitrin-Neo***	1.29±0.06a	0.84±0.06b	1.38±0.08a	1.39±0.02a
***Spinacetin-Glu-HMG-Glu***	6.5±0.21b	3.31±0.32c	6.19±0.29b	7.35±0.29a
***Limocitrin-Glu-HMG-Glu***	6.51±0.25a	3.39±0.26b	6.03±0.11a	6.11±0.32a
***Isorhamnetin-3-O-Neo***	1.31±0.04a, b	1.13±0.08b	1.63±0.25a	1.41±0.04a, b
***Limocitrol-Glu-HMG***	5.39±0.15a	3.98±0.27b	5±0.21a	5.03±0.34a
***Limocitrin-HMG-Glu***	4.29±0.11a	2.71±0.19b	4.22±0.24a	4.17±0.16a
***Quercetin-3-O-Neo***	0.48±0.02c	0.85±0.03b	4.35±0.28a	4.08±0.05a

Glu, glucoside; Neo, neohesperidoside; Rut, rutinoside; HMG, 3-hydroxy-3-methyl-glutaryl.

## Conclusions

The present study shows a clear discrimination among flavonoid profiles in extracts from lemon as a function of sample pretreatment and auxiliary energy applied for improving extraction. Sample dehydration prior to flavonoids extraction provided better results than fresh samples. Despite of both lyophilized and air-dried samples allow obtaining extracts with higher amounts of flavonoids, lyophilization decreases or avoids the undesirable reactions produced by high temperature. On the other hand, it has been demonstrated that the USAE method was the best to extract flavonoids, showing higher yields than all other methods in a shorter time. In addition, MAE proved to be better than SE to extract flavonoids, thanks to the thermostability of these compounds [27]. Despite MAE could be a good alternative to extract the target metabolites, the effect of temperature on the extraction of other citrus components should be taken into account. Finally, SHLE showed to be the less favorable to extract flavonoids from lemon. Despite of the fact that flavonoids are no thermolabile, the large amount of pectin in the fruit causes interference and makes difficult to obtain the extract [22]; however, for rough materials like wood SHLE has proved be a good alternative to conventional extraction methods [23].

## Supporting Information

S1 Table Peak area, mean, standard deviation (SD) and relative standard deviation (RSD, %), for the analyzed samples used to evaluate the effect of sample pretreatment on the extraction of flavonoids from lemon. (DOCX)

S2 Table Peak area, mean, standard deviation (SD) and relative standard deviation (RSD, %), for the analyzed samples used to evaluate the effect of auxiliary energies on the extraction of flavonoids from lemon. (DOCX)

## Abbreviations

ACN: acetonitrile
LC: liquid chromatography
QTOF: quadrupole–time of flight detector
MS: mass spectrometry
ESI: electrospray ionization source
RT: retention time
SHLE: superheated liquid extraction
MAE: microwave-assisted extraction
USAE: ultrasound-assisted extraction
SE: shaking extraction method
PCA: principal component analysis
Glu: glucoside
Neo: neohesperidoside
Rut: rutinoside
HMG: 3-hydroxy-3-methyl-glutaryl
