# Supplementary material for: Comparative Study of the Effect of Sample Pretreatment and Extraction on the Determination of Flavonoids from Lemon (Citrus limon)
Source: PLoS One. 2016 Jan 25;11(1):e0148056. doi: 10.1371/journal.pone.0148056 (PMC4726533; doi:10.1371/journal.pone.0148056)
Supplement: S1 Table — (DOCX) [file pone.0148056.s001.docx]

**Supplementary table 1:** Peak area, mean, standard deviation (SD) and relative standard deviation (RSD, %), for the analyzed samples used to evaluate the effect of sample pretreatment on the extraction of flavonoids from lemon.

| **Shaking extraction, lyophilized samples** | | | | | | | |
| --- | --- | --- | --- | --- | --- | --- | --- |
| **Compound name** | **Lyo-S01** | **Lyo-S02** | **Lyo-S03** | **Lyo-S04** | **Mean** | **SD** | **RSD** |
| **Neoeriocitrin** | 113907048 | 116856816 | 118593056 | 122576848 | 117983442 | 3136769 | 2.66 |
| **Neohesperidin** | 109591144 | 115957080 | 118965480 | 124804792 | 117329624 | 5484572 | 4.67 |
| **Eriodictyol-Glu-Rha-Glu** | 5608255 | 6081195 | 6549743 | 6502100 | 6185323 | 379802 | 6.14 |
| **Naringin** | 4202361 | 4398034 | 4677751 | 4760195 | 4509585 | 222438 | 4.93 |
| **Hesperetin** | 3426701 | 3837885 | 3629472 | 3656310 | 3637592 | 145781 | 4.01 |
| **Hesperetin-7-O-Rha** | 6027480 | 8133915 | 6303126 | 7645912 | 7027608 | 884780 | 12.59 |
| **Eriocitrin** | 580225 | 716472 | 616951 | 643243 | 639223 | 49901 | 7.81 |
| **Hesperidin** | 1356317 | 1389609 | 1420065 | 1491625 | 1414404 | 49960 | 3.53 |
| **Eriodictyol-Neo-Rha** | 395825 | 479836 | 434006 | 394651 | 426079 | 34841 | 8.18 |
| **Luteolin-Neo** | 20820488 | 20737128 | 22324520 | 22082432 | 21491142 | 718063 | 3.34 |
| **Diosmetin-Glu** | 7471476 | 7211628 | 8028783 | 7955071 | 7666740 | 338919 | 4.42 |
| **Luteolin-Glu-Rha** | 19845814 | 20424712 | 21181284 | 23360320 | 21203033 | 1332496 | 6.28 |
| **Rhoifolin** | 3319204 | 3451424 | 3937506 | 3860239 | 3642093 | 262425 | 7.21 |
| **Neodiosmin** | 21134626 | 26590936 | 21611164 | 26682164 | 24004723 | 2637412 | 10.99 |
| **Vitexin -O-xyloside** | 2863315 | 3025613 | 2986001 | 3032132 | 2976765 | 67838 | 2.28 |
| **Diosmin** | 730242 | 697634 | 764588 | 741897 | 733590 | 24155 | 3.29 |
| **Vitexin** | 1151957 | 1124202 | 1313383 | 1311871 | 1225353 | 87825 | 7.17 |
| **Apigenin-Glu-Rha-Glu** | 1493416 | 1523874 | 1660805 | 1654653 | 1583187 | 75347 | 4.76 |
| **Diosmetin-Glu-Rha** | 870897 | 893521 | 896521 | 924495 | 896359 | 19026 | 2.12 |
| **Luteolin-Rut-Glu** | 1152455 | 1187066 | 1312053 | 1358454 | 1252507 | 85240 | 6.81 |
| **Vitexin-2-Rha** | 669381 | 694938 | 769653 | 755923 | 722474 | 41599 | 5.76 |
| **Homoorientin** | 1176574 | 1133776 | 1358817 | 1175656 | 1211206 | 86959 | 7.18 |
| **Orientin** | 637200 | 596097 | 818479 | 747009 | 699696 | 88015 | 12.58 |
| **Quercetin-Glu-Rha-Glu** | 502249 | 536463 | 553413 | 588548 | 545168 | 31095 | 5.70 |
| **Rutin** | 2357987 | 2453883 | 2461539 | 2545542 | 2454738 | 66432 | 2.71 |
| **Limocitrin-Neo** | 1251164 | 1211915 | 1327227 | 1354538 | 1286211 | 57226 | 4.45 |
| **Spinacetin-Glu-HMG-Glu** | 6171914 | 6523308 | 6554026 | 6753257 | 6500626 | 209308 | 3.22 |
| **Limocitrin-Glu-HMG-Glu** | 6314008 | 6265655 | 6560163 | 6903497 | 6510831 | 252721 | 3.88 |
| **Isorhamnetin-3-O-Neo** | 1279628 | 1264338 | 1338933 | 1363997 | 1311724 | 41074 | 3.13 |
| **Limocitrol-Glu-HMG** | 5193403 | 5369331 | 5387842 | 5623164 | 5393435 | 152807 | 2.83 |
| **Limocitrin-HMG-Glu** | 4181047 | 4198638 | 4315560 | 4464175 | 4289855 | 113146 | 2.64 |
| **Quercetin-3-O-Neo** | 471765 | 457332 | 472824 | 512697 | 478655 | 20585 | 4.30 |
| **Shaking extraction, fresh samples** | | | | | | | |
| **Compound name** | **Fresh-S01** | **Fresh-S02** | **Fresh-S03** | **Fresh-S04** | **Mean** | **SD** | **RSD** |
| **Neoeriocitrin** | 98783880 | 101946280 | 87352600 | 89363184 | 94361486 | 6148052 | 6.52 |
| **Neohesperidin** | 58707408 | 61367776 | 61689832 | 63106744 | 61217940 | 1590242 | 2.60 |
| **Eriodictyol-Glu-Rha-Glu** | 4395884 | 4668131 | 4035307 | 3978031 | 4269338 | 280482 | 6.57 |
| **Naringin** | 4066290 | 4122596 | 3831461 | 3889143 | 3977373 | 120489 | 3.03 |
| **Hesperetin** | 1950586 | 2119659 | 1970493 | 2128877 | 2042404 | 82231 | 4.03 |
| **Hesperetin-7-O-Rha** | 3315539 | 3848170 | 3461414 | 3919471 | 3636149 | 254238 | 6.99 |
| **Eriocitrin** | 625196 | 560506 | 307462 | 493453 | 496654 | 118748 | 23.91 |
| **Hesperidin** | 1331784 | 1351755 | 1240269 | 1319296 | 1310776 | 42322 | 3.23 |
| **Eriodictyol-Neo-Rha** | 354719 | 372515 | 284060 | 293357 | 326163 | 38121 | 11.69 |
| **Luteolin-Neo** | 19443476 | 19885200 | 17509704 | 17969220 | 18701900 | 988469 | 5.29 |
| **Diosmetin-Glu** | 6891051 | 6700217 | 6271148 | 6199426 | 6515461 | 289296 | 4.44 |
| **Luteolin-Glu-Rha** | 9585247 | 10198664 | 9741618 | 10053007 | 9894634 | 243234 | 2.46 |
| **Rhoifolin** | 3012060 | 2995449 | 3010141 | 3143676 | 3040332 | 60011 | 1.97 |
| **Neodiosmin** | 4835753 | 4865287 | 4879385 | 5096291 | 4919179 | 103461 | 2.10 |
| **Vitexin -O-xyloside** | 2252109 | 2332853 | 1884621 | 1969209 | 2109698 | 187401 | 8.88 |
| **Diosmin** | 895902 | 1058761 | 964902 | 857139 | 944176 | 76592 | 8.11 |
| **Vitexin** | 965872 | 839243 | 889433 | 909062 | 900902 | 45335 | 5.03 |
| **Apigenin-Glu-Rha-Glu** | 864694 | 855479 | 698441 | 716015 | 783657 | 76751 | 9.79 |
| **Diosmetin-Glu-Rha** | 347614 | 394735 | 365420 | 370007 | 369444 | 16828 | 4.55 |
| **Luteolin-Rut-Glu** | 496939 | 493855 | 474629 | 479843 | 486317 | 9330 | 1.92 |
| **Vitexin-2-Rha** | 431434 | 444715 | 357489 | 367320 | 400240 | 38283 | 9.57 |
| **Homoorientin** | 903551 | 1174830 | 966437 | 847746 | 973141 | 123784 | 12.72 |
| **Orientin** | 614936 | 629535 | 547216 | 738260 | 632487 | 68513 | 10.83 |
| **Quercetin-Glu-Rha-Glu** | 930618 | 928589 | 884660 | 914369 | 914559 | 18362 | 2.01 |
| **Rutin** | 4160572 | 4344091 | 3634166 | 3835810 | 3993660 | 276050 | 6.91 |
| **Limocitrin-Neo** | 1097341 | 1061615 | 1098283 | 1120770 | 1094502 | 21177 | 1.93 |
| **Spinacetin-Glu-HMG-Glu** | 5481979 | 5346332 | 5807091 | 5916069 | 5637868 | 232017 | 4.12 |
| **Limocitrin-Glu-HMG-Glu** | 5460218 | 5374118 | 5723793 | 5824608 | 5595684 | 184567 | 3.30 |
| **Isorhamnetin-3-O-Neo** | 1919556 | 1874201 | 1756368 | 1754654 | 1826195 | 72482 | 3.97 |
| **Limocitrol-Glu-HMG** | 18400864 | 18937168 | 21993904 | 22646480 | 20494604 | 1849853 | 9.03 |
| **Limocitrin-HMG-Glu** | 12242077 | 12086575 | 14520051 | 15025591 | 13468574 | 1317585 | 9.78 |
| **Quercetin-3-O-Neo** | 249479 | 251273 | 224056 | 228387 | 238299 | 12190 | 5.12 |
| **Shaking extraction, air-drying samples** | | | | | | | |
| **Compound name** | **Air-S01** | **Air-S02** | **Air-S03** | **Air-S04** | **Mean** | **SD** | **RSD** |
| **Neoeriocitrin** | 108837384 | 112196736 | 110214240 | 114678624 | 111481746 | 2198321 | 1.97 |
| **Neohesperidin** | 40979428 | 42045376 | 42850556 | 44247652 | 42530753 | 1192918 | 2.80 |
| **Eriodictyol-Glu-Rha-Glu** | 5359929 | 5518884 | 5588657 | 5747068 | 5553635 | 139080 | 2.50 |
| **Naringin** | 4767473 | 4906339 | 5015051 | 5196031 | 4971224 | 156671 | 3.15 |
| **Hesperetin** | 1227639 | 1530597 | 1245019 | 1527772 | 1382757 | 146560 | 10.60 |
| **Hesperetin-7-O-Rha** | 2118990 | 2815534 | 2192190 | 2840295 | 2491752 | 337271 | 13.54 |
| **Eriocitrin** | 949165 | 673186 | 687959 | 659516 | 742457 | 119766 | 16.13 |
| **Hesperidin** | 1543938 | 1585584 | 1703398 | 1759496 | 1648104 | 86927 | 5.27 |
| **Eriodictyol-Neo-Rha** | 376597 | 390272 | 369301 | 411344 | 386879 | 16006 | 4.14 |
| **Luteolin-Neo** | 22722914 | 22910024 | 25438628 | 26404564 | 24369033 | 1591056 | 6.53 |
| **Diosmetin-Glu** | 8215976 | 8079115 | 9184687 | 9004342 | 8621030 | 480203 | 5.57 |
| **Luteolin-Glu-Rha** | 7551880 | 8319499 | 8002701 | 9004787 | 8219717 | 529007 | 6.44 |
| **Rhoifolin** | 2766379 | 2699592 | 3022189 | 2990452 | 2869653 | 139146 | 4.85 |
| **Neodiosmin** | 2709104 | 2958453 | 2632008 | 2808900 | 2777116 | 122040 | 4.39 |
| **Vitexin -O-xyloside** | 2492641 | 2521094 | 2752650 | 2834974 | 2650340 | 146740 | 5.54 |
| **Diosmin** | 1194953 | 1180354 | 1340706 | 1296370 | 1253096 | 67491 | 5.39 |
| **Vitexin** | 1361697 | 1385052 | 1480992 | 1496528 | 1431067 | 58539 | 4.09 |
| **Apigenin-Glu-Rha-Glu** | 731952 | 739694 | 779530 | 782319 | 758374 | 22738 | 3.00 |
| **Diosmetin-Glu-Rha** | 623514 | 561640 | 512563 | 510742 | 552115 | 46002 | 8.33 |
| **Luteolin-Rut-Glu** | 517585 | 508624 | 537680 | 507802 | 517923 | 12035 | 2.32 |
| **Vitexin-2-Rha** | 466026 | 466797 | 520774 | 537377 | 497744 | 31879 | 6.40 |
| **Homoorientin** | 1049332 | 969972 | 1100621 | 971194 | 1022780 | 55259 | 5.40 |
| **Orientin** | 917331 | 578524 | 928692 | 669360 | 773477 | 152997 | 19.78 |
| **Quercetin-Glu-Rha-Glu** | 901100 | 964986 | 1094569 | 1141596 | 1025563 | 96677 | 9.43 |
| **Rutin** | 4560446 | 4941591 | 5136501 | 5312868 | 4987852 | 279530 | 5.60 |
| **Limocitrin-Neo** | 1361613 | 1311239 | 1565342 | 1520040 | 1439559 | 105877 | 7.35 |
| **Spinacetin-Glu-HMG-Glu** | 6084864 | 6417886 | 7381395 | 7506286 | 6847608 | 609349 | 8.90 |
| **Limocitrin-Glu-HMG-Glu** | 6466312 | 6533959 | 7669930 | 7832166 | 7125592 | 628536 | 8.82 |
| **Isorhamnetin-3-O-Neo** | 2157080 | 2227478 | 2521881 | 2527190 | 2358407 | 167993 | 7.12 |
| **Limocitrol-Glu-HMG** | 22043100 | 23206972 | 25640076 | 27160278 | 24512607 | 2005274 | 8.18 |
| **Limocitrin-HMG-Glu** | 15439678 | 15696738 | 18121232 | 18412604 | 16917563 | 1356330 | 8.02 |
| **Quercetin-3-O-Neo** | 242140 | 283354 | 205179 | 220456 | 237782 | 29406 | 12.37 |
