# Supplementary material for: Comparative Study of the Effect of Sample Pretreatment and Extraction on the Determination of Flavonoids from Lemon (Citrus limon)
Source: PLoS One. 2016 Jan 25;11(1):e0148056. doi: 10.1371/journal.pone.0148056 (PMC4726533; doi:10.1371/journal.pone.0148056)
Supplement: S2 Table — (DOCX) [file pone.0148056.s002.docx]

**Supplementary table 2:** Peak area, mean, standard deviation (SD) and relative standard deviation (RSD, %), for the analyzed samples used to evaluate the effect of auxiliary energies on the extraction of flavonoids from lemon.

| **Shaking extraction, lyophilized samples** | | | | | | | |
| --- | --- | --- | --- | --- | --- | --- | --- |
| **Compound Name** | **Shaking-01** | **Shaking-02** | **Shaking-03** | **Shaking-04** | **Mean** | **SD** | **RSD** |
| **Quercetin-Glu-Rha-Glu** | 502249.00 | 536463.00 | 553413.00 | 588548.00 | 545168.25 | 31095.10 | 5.70 |
| **Rutin** | 2357987.00 | 2453883.00 | 2461539.00 | 2545542.00 | 2454737.75 | 66432.50 | 2.71 |
| **Limocitrin-Neo** | 1251164.00 | 1211915.00 | 1327227.00 | 1354538.00 | 1286211.00 | 57225.60 | 4.45 |
| **Spinacetin-Glu-HMG-Glu** | 6171914.00 | 6523308.00 | 6554026.00 | 6753257.00 | 6500626.25 | 209308.41 | 3.22 |
| **Limocitrin-Glu-HMG-Glu** | 6314008.00 | 6265655.00 | 6560163.00 | 6903497.00 | 6510830.75 | 252720.60 | 3.88 |
| **Isorhamnetin-3-O-Neo** | 1279628.00 | 1264338.00 | 1338933.00 | 1363997.00 | 1311724.00 | 41074.27 | 3.13 |
| **Limocitrol-Glu-HMG** | 5193403.00 | 5369331.00 | 5387842.00 | 5623164.00 | 5393435.00 | 152807.48 | 2.83 |
| **Limocitrin-HMG-Glu** | 4181047.00 | 4198638.00 | 4315560.00 | 4464175.00 | 4289855.00 | 113145.85 | 2.64 |
| **Quercetin-3-O-Neo** | 471765.00 | 457332.00 | 472824.00 | 512697.00 | 478654.50 | 20585.19 | 4.30 |
| **Eriocitrin** | 580224.00 | 716472.00 | 616955.00 | 643243.00 | 639223.50 | 49900.69 | 7.81 |
| **Eriodictyol-Glu-Rha-Glu** | 5608255.00 | 6081195.00 | 6549743.00 | 6502100.00 | 6185323.25 | 379802.49 | 6.14 |
| **Eriodictyol-Neo-Rha** | 395825.44 | 479835.87 | 434005.60 | 394650.99 | 426079.48 | 34841.17 | 8.18 |
| **Neoeriocitrin** | 113907048.00 | 116856816.00 | 118593056.00 | 122576848.00 | 117983442.00 | 3136769.12 | 2.66 |
| **Naringin** | 4202361.00 | 4398034.00 | 4677751.00 | 4760195.00 | 4509585.25 | 222438.41 | 4.93 |
| **Hesperidin** | 1356317.00 | 1389609.00 | 1420065.00 | 1491625.00 | 1414404.00 | 49960.04 | 3.53 |
| **Hesperetin** | 3426701.00 | 3837885.00 | 3629472.00 | 3656310.00 | 3637592.00 | 145781.17 | 4.01 |
| **Hesperetin-7-O-Rha** | 6027480.00 | 8133915.00 | 6303126.00 | 7645912.00 | 7027608.25 | 884780.37 | 12.59 |
| **Neohesperidin** | 109591144.00 | 115957080.00 | 118965480.00 | 124804792.00 | 117329624.00 | 5484571.80 | 4.67 |
| **Apigenin-Glu-Rha-Glu** | 1493416.00 | 1523874.00 | 1660805.00 | 1654653.00 | 1583187.00 | 75347.21 | 4.76 |
| **Luteolin-Rut-Glu** | 1152455.00 | 1187066.00 | 1312053.00 | 1358454.00 | 1252507.00 | 85239.98 | 6.81 |
| **Homoorientin** | 1176574.00 | 1133776.00 | 1358817.00 | 1175656.00 | 1211205.75 | 86959.17 | 7.18 |
| **Orientin** | 637200.19 | 596097.04 | 818479.17 | 747009.42 | 699696.46 | 88014.87 | 12.58 |
| **Vitexin -O-xyloside** | 2863315.00 | 3025613.00 | 2986001.00 | 3032132.00 | 2976765.25 | 67837.74 | 2.28 |
| **Vitexin** | 1151957.15 | 1124201.77 | 1313382.89 | 1311871.27 | 1225353.27 | 87825.39 | 7.17 |
| **Vitexin-2-Rha** | 669381.08 | 694937.89 | 769653.35 | 755922.99 | 722473.83 | 41598.74 | 5.76 |
| **Luteolin-Glu-Rha** | 2665601.00 | 2817620.00 | 3046805.00 | 2978981.00 | 2877251.75 | 147859.00 | 5.14 |
| **Luteolin-Neo** | 19845814.00 | 20424712.00 | 21181284.00 | 23360320.00 | 21203032.50 | 1332496.46 | 6.28 |
| **Diosmetin-Glu** | 5209934.00 | 5158569.00 | 5649361.00 | 5384995.00 | 5350714.75 | 191770.65 | 3.58 |
| **Rhoifolin** | 3319204.00 | 3451424.00 | 3937506.00 | 3860239.00 | 3642093.25 | 262425.46 | 7.21 |
| **Diosmin** | 730242.00 | 697634.00 | 764588.00 | 741897.00 | 733590.25 | 24155.36 | 3.29 |
| **Neodiosmin** | 21134626.00 | 26590936.00 | 21611164.00 | 26682164.00 | 24004722.50 | 2637412.06 | 10.99 |
| **Diosmetin-Glu-Rha** | 870897.00 | 893521.00 | 896521.00 | 924495.00 | 896358.50 | 19026.48 | 2.12 |
| **Ultrasound assisted extraction, lyophilized samples** | | | | | | | |
| **Compound Name** | **USAE-01** | **USAE-02** | **USAE-03** | **USAE-04** | **Mean** | **SD** | **RSD** |
| **Quercetin-Glu-Rha-Glu** | 690252.00 | 568024.00 | 718715.00 | 707276.00 | 671066.75 | 60347.63 | 8.99 |
| **Rutin** | 2399778.00 | 2580085.00 | 2391993.00 | 2436003.00 | 2451964.75 | 75811.68 | 3.09 |
| **Limocitrin-Neo** | 1373825.00 | 1424299.00 | 1375547.00 | 1397938.00 | 1392902.25 | 20471.08 | 1.47 |
| **Spinacetin-Glu-HMG-Glu** | 7423161.00 | 6957210.00 | 7761785.00 | 7263240.00 | 7351349.00 | 290139.37 | 3.95 |
| **Limocitrin-Glu-HMG-Glu** | 6348886.00 | 5708809.00 | 6490884.00 | 5895295.00 | 6110968.50 | 319838.96 | 5.23 |
| **Isorhamnetin-3-O-Neo** | 1449541.00 | 1389986.00 | 1447306.00 | 1369789.00 | 1414155.50 | 35013.00 | 2.48 |
| **Limocitrol-Glu-HMG** | 5132356.00 | 4997250.00 | 5467436.00 | 4522335.00 | 5029844.25 | 339346.14 | 6.75 |
| **Limocitrin-HMG-Glu** | 4259054.00 | 4001987.00 | 4384172.00 | 4041599.00 | 4171703.00 | 156926.61 | 3.76 |
| **Quercetin-3-O-Neo** | 4133334.00 | 4015053.00 | 4069211.00 | 4111256.00 | 4082213.50 | 45100.91 | 1.10 |
| **Eriocitrin** | 869022.00 | 847524.00 | 845495.00 | 800547.00 | 840647.00 | 24919.61 | 2.96 |
| **Eriodictyol-Glu-Rha-Glu** | 7842555.00 | 7273931.00 | 8267646.00 | 8130677.00 | 7878702.25 | 381386.75 | 4.84 |
| **Eriodictyol-Neo-Rha** | 540998.96 | 572813.85 | 646078.51 | 623903.96 | 595948.82 | 41379.92 | 6.94 |
| **Neoeriocitrin** | 147277824.00 | 142610672.00 | 139524352.00 | 153069088.00 | 145620484.00 | 5110045.77 | 3.51 |
| **Naringin** | 6419988.00 | 5346686.00 | 6759674.00 | 6343745.00 | 6217523.25 | 526594.92 | 8.47 |
| **Hesperidin** | 1842710.00 | 1778443.00 | 1884150.00 | 1930814.00 | 1859029.25 | 56001.26 | 3.01 |
| **Hesperetin** | 6930689.00 | 7127338.00 | 7252548.00 | 7158203.00 | 7117194.50 | 117142.50 | 1.65 |
| **Hesperetin-7-O-Rha** | 14268684.00 | 14366354.00 | 15855096.00 | 14915256.00 | 14851347.50 | 629744.15 | 4.24 |
| **Neohesperidin** | 229733568.00 | 223753872.00 | 223714768.00 | 249294496.00 | 231624176.00 | 10491842.40 | 4.53 |
| **Apigenin-Glu-Rha-Glu** | 2822147.00 | 2999803.00 | 3122976.00 | 3430234.00 | 3093790.00 | 221737.38 | 7.17 |
| **Luteolin-Rut-Glu** | 2033595.00 | 2188407.00 | 2356630.00 | 2477924.00 | 2264139.00 | 168184.78 | 7.43 |
| **Homoorientin** | 1122144.00 | 1018166.00 | 1092012.00 | 998342.00 | 1057666.00 | 51030.99 | 4.82 |
| **Orientin** | 773273.38 | 692712.90 | 776952.84 | 681065.19 | 731001.08 | 44322.93 | 6.06 |
| **Vitexin -O-xyloside** | 3225068.00 | 3054736.00 | 3221393.00 | 2805642.00 | 3076709.75 | 170956.12 | 5.56 |
| **Vitexin** | 1377344.53 | 1377063.72 | 1356013.40 | 1206485.77 | 1329226.86 | 71390.77 | 5.37 |
| **Vitexin-2-Rha** | 885825.44 | 864619.32 | 893081.77 | 791849.68 | 858844.05 | 40068.12 | 4.67 |
| **Luteolin-Glu-Rha** | 6108829.00 | 6531121.00 | 6757890.00 | 6410448.00 | 6452072.00 | 234158.98 | 3.63 |
| **Luteolin-Neo** | 38900292.00 | 38653816.00 | 39266912.00 | 39856448.00 | 39169367.00 | 452711.14 | 1.16 |
| **Diosmetin-Glu** | 5487854.00 | 4800354.00 | 5776095.00 | 4806945.00 | 5217812.00 | 426522.41 | 8.17 |
| **Rhoifolin** | 9160867.00 | 9667067.00 | 10299182.00 | 11416778.00 | 10135973.50 | 842289.02 | 8.31 |
| **Diosmin** | 851808.00 | 699927.00 | 908613.00 | 766630.00 | 806744.50 | 79729.33 | 9.88 |
| **Neodiosmin** | 45937356.00 | 47217788.00 | 48778540.00 | 49701032.00 | 47908679.00 | 1443314.95 | 3.01 |
| **Diosmetin-Glu-Rha** | 4476051.00 | 3718737.00 | 4238815.00 | 3835135.00 | 4067184.50 | 304914.53 | 7.50 |
| **Microwave assisted extraction, lyophilized samples** | | | | | | | |
| **Compound Name** | **MAE-01** | **MAE-02** | **MAE-03** | **MAE-04** | **Mean** | **SD** | **RSD** |
| **Quercetin-Glu-Rha-Glu** | 560225.00 | 615128.00 | 767419.00 | 882223.00 | 706248.75 | 126821.39 | 17.96 |
| **Rutin** | 2532433.00 | 2529267.00 | 2963835.00 | 2801539.00 | 2706768.50 | 185043.43 | 6.84 |
| **Limocitrin-Neo** | 1302343.00 | 1299925.00 | 1426834.00 | 1476756.00 | 1376464.50 | 77375.32 | 5.62 |
| **Spinacetin-Glu-HMG-Glu** | 6333287.00 | 6564520.00 | 6046749.00 | 5810346.00 | 6188725.50 | 285240.21 | 4.61 |
| **Limocitrin-Glu-HMG-Glu** | 6214707.00 | 5972831.00 | 5993051.00 | 5948268.00 | 6032214.25 | 106548.94 | 1.77 |
| **Isorhamnetin-3-O-Neo** | 1397048.00 | 1361480.00 | 1875386.00 | 1873669.00 | 1626895.75 | 247951.58 | 15.24 |
| **Limocitrol-Glu-HMG** | 4670760.00 | 5023560.00 | 5259573.00 | 5049938.00 | 5000957.75 | 211436.95 | 4.23 |
| **Limocitrin-HMG-Glu** | 3807111.00 | 4335906.00 | 4313592.00 | 4404626.00 | 4215308.75 | 238049.22 | 5.65 |
| **Quercetin-3-O-Neo** | 4320724.00 | 4123800.00 | 4804696.00 | 4137671.00 | 4346722.75 | 275596.00 | 6.34 |
| **Eriocitrin** | 748658.00 | 764329.00 | 721649.00 | 677144.00 | 727945.00 | 33065.11 | 4.54 |
| **Eriodictyol-Glu-Rha-Glu** | 6704269.00 | 6484133.00 | 5370086.00 | 5710802.00 | 6067322.50 | 546048.86 | 9.00 |
| **Eriodictyol-Neo-Rha** | 507889.61 | 511971.02 | 546657.53 | 497091.97 | 515902.53 | 18569.94 | 3.60 |
| **Neoeriocitrin** | 123526432.00 | 121289464.00 | 131992408.00 | 140259392.00 | 129266924.00 | 7497595.15 | 5.80 |
| **Naringin** | 5087363.00 | 5121449.00 | 6352113.00 | 7220639.00 | 5945391.00 | 895373.21 | 15.06 |
| **Hesperidin** | 1556522.00 | 1611467.00 | 2017415.00 | 2256898.00 | 1860575.50 | 289902.45 | 15.58 |
| **Hesperetin** | 7755492.00 | 7320453.00 | 7959439.00 | 7110252.00 | 7536409.00 | 337342.01 | 4.48 |
| **Hesperetin-7-O-Rha** | 15433390.00 | 15836233.00 | 16761465.00 | 15702736.00 | 15933456.00 | 499587.06 | 3.14 |
| **Neohesperidin** | 185658096.00 | 185727424.00 | 199550864.00 | 214767072.00 | 196425864.00 | 12005904.61 | 6.11 |
| **Apigenin-Glu-Rha-Glu** | 2201202.00 | 2057690.00 | 2061356.00 | 2469551.00 | 2197449.75 | 167412.27 | 7.62 |
| **Luteolin-Rut-Glu** | 1631063.00 | 1460851.00 | 1443364.00 | 1582156.00 | 1529358.50 | 79403.58 | 5.19 |
| **Homoorientin** | 1095263.00 | 1236423.00 | 1364061.00 | 1313516.00 | 1252315.75 | 101428.07 | 8.10 |
| **Orientin** | 773730.22 | 688807.17 | 822678.96 | 768057.49 | 763318.46 | 47975.16 | 6.29 |
| **Vitexin -O-xyloside** | 3234032.00 | 3258788.00 | 2609881.00 | 2661693.00 | 2941098.50 | 305985.75 | 10.40 |
| **Vitexin** | 1161330.50 | 1191767.17 | 1425409.97 | 1399682.36 | 1294547.50 | 118836.96 | 9.18 |
| **Vitexin-2-Rha** | 727636.26 | 796564.64 | 719547.52 | 756151.61 | 749975.01 | 30139.66 | 4.02 |
| **Luteolin-Glu-Rha** | 4692324.00 | 4029106.00 | 4478464.00 | 4665667.00 | 4466390.25 | 265575.12 | 5.95 |
| **Luteolin-Neo** | 30296104.00 | 27314788.00 | 26884346.00 | 27767868.00 | 28065776.50 | 1325035.09 | 4.72 |
| **Diosmetin-Glu** | 5296313.00 | 5450252.00 | 6229317.00 | 6065282.00 | 5760291.00 | 395096.40 | 6.86 |
| **Rhoifolin** | 5982989.00 | 5562695.00 | 6591062.00 | 5996032.00 | 6033194.50 | 366226.56 | 6.07 |
| **Diosmin** | 748713.00 | 747495.00 | 566621.00 | 749281.00 | 703027.50 | 78756.97 | 11.20 |
| **Neodiosmin** | 45770672.00 | 47180052.00 | 42664422.00 | 47145272.00 | 45690104.50 | 1837028.97 | 4.02 |
| **Diosmetin-Glu-Rha** | 8821761.00 | 9594041.00 | 8335000.00 | 8990680.00 | 8935370.50 | 450071.61 | 5.04 |
| **Superheated liquid extraction, lyophilized samples** | | | | | | | |
| **Compound Name** | **SHLE-01** | **SHLE-02** | **SHLE-03** | **SHLE-04** | **Mean** | **SD** | **RSD** |
| **Quercetin-Glu-Rha-Glu** | 393386.00 | 421603.00 | 366899.00 | 465858.00 | 411936.50 | 36651.97 | 8.90 |
| **Rutin** | 1676154.00 | 1840330.00 | 1832382.00 | 1982859.00 | 1832931.25 | 108527.05 | 5.92 |
| **Limocitrin-Neo** | 760766.00 | 816852.00 | 828193.00 | 934400.00 | 835052.75 | 62782.80 | 7.52 |
| **Spinacetin-Glu-HMG-Glu** | 3050436.00 | 3467813.00 | 2964656.00 | 3746966.00 | 3307467.75 | 317196.52 | 9.59 |
| **Limocitrin-Glu-HMG-Glu** | 3120468.00 | 3222383.00 | 3410042.00 | 3799168.00 | 3388015.25 | 259107.76 | 7.65 |
| **Isorhamnetin-3-O-Neo** | 1031860.00 | 1124533.00 | 1100214.00 | 1260837.00 | 1129361.00 | 83164.36 | 7.36 |
| **Limocitrol-Glu-HMG** | 3634424.00 | 3899916.00 | 3988484.00 | 4380974.00 | 3975949.50 | 267685.79 | 6.73 |
| **Limocitrin-HMG-Glu** | 2504782.00 | 2637029.00 | 2660876.00 | 3022329.00 | 2706254.00 | 191928.13 | 7.09 |
| **Quercetin-3-O-Neo** | 830908.00 | 859105.00 | 821483.00 | 895083.00 | 851644.75 | 28645.60 | 3.36 |
| **Eriocitrin** | 313527.00 | 321270.00 | 360605.00 | 326060.00 | 330365.50 | 18022.40 | 5.46 |
| **Eriodictyol-Glu-Rha-Glu** | 2426831.00 | 2927989.00 | 2737456.00 | 3127801.00 | 2805019.25 | 258312.20 | 9.21 |
| **Eriodictyol-Neo-Rha** | 281354.88 | 240119.30 | 304962.18 | 215834.46 | 260567.71 | 34720.95 | 13.33 |
| **Neoeriocitrin** | 85098224.00 | 86841104.00 | 87001736.00 | 92089512.00 | 87757644.00 | 2610030.54 | 2.97 |
| **Naringin** | 3083187.00 | 3375340.00 | 3388093.00 | 3621570.00 | 3367047.50 | 190964.76 | 5.67 |
| **Hesperidin** | 1418178.00 | 1542048.00 | 1403595.00 | 1599970.00 | 1490947.75 | 82799.50 | 5.55 |
| **Hesperetin** | 3610496.00 | 3532908.00 | 3448077.00 | 3255969.00 | 3461862.50 | 132024.20 | 3.81 |
| **Hesperetin-7-O-Rha** | 6256321.00 | 7905391.00 | 6074346.00 | 7310726.00 | 6886696.00 | 754126.33 | 10.95 |
| **Neohesperidin** | 102342216.00 | 111222928.00 | 95141776.00 | 104894864.00 | 103400446.00 | 5760859.11 | 5.57 |
| **Apigenin-Glu-Rha-Glu** | 1253153.00 | 1485142.00 | 1129834.00 | 1286567.00 | 1288674.00 | 127569.59 | 9.90 |
| **Luteolin-Rut-Glu** | 650178.00 | 682702.00 | 622772.00 | 675323.00 | 657743.75 | 23517.06 | 3.58 |
| **Homoorientin** | 706585.00 | 671558.00 | 687446.00 | 624812.00 | 672600.25 | 30249.64 | 4.50 |
| **Orientin** | 301907.17 | 344628.25 | 281969.24 | 297535.44 | 306510.03 | 23221.64 | 7.58 |
| **Vitexin -O-xyloside** | 1245400.00 | 1314349.00 | 1137828.00 | 1263922.00 | 1240374.75 | 64357.89 | 5.19 |
| **Vitexin** | 447416.81 | 527405.11 | 541231.48 | 602423.06 | 529619.12 | 55220.88 | 10.43 |
| **Vitexin-2-Rha** | 320116.69 | 340184.14 | 297442.13 | 316436.57 | 318544.88 | 15169.86 | 4.76 |
| **Luteolin-Glu-Rha** | 1834878.00 | 1832309.00 | 1987776.00 | 1872447.00 | 1881852.50 | 63185.07 | 3.36 |
| **Luteolin-Neo** | 12651309.00 | 13349440.00 | 13089063.00 | 13154676.00 | 13061122.00 | 255248.42 | 1.95 |
| **Diosmetin-Glu** | 2960880.00 | 3007006.00 | 3062370.00 | 3032190.00 | 3015611.50 | 37184.66 | 1.23 |
| **Rhoifolin** | 2941008.00 | 3431959.00 | 2599430.00 | 2826916.00 | 2949828.25 | 304308.32 | 10.32 |
| **Diosmin** | 494346.00 | 523491.00 | 428572.00 | 482142.00 | 482137.75 | 34381.76 | 7.13 |
| **Neodiosmin** | 14488795.00 | 16940284.00 | 14529763.00 | 14941298.00 | 15225035.00 | 1005986.87 | 6.61 |
| **Diosmetin-Glu-Rha** | 2087844.00 | 2883375.00 | 2231860.00 | 2333558.00 | 2384159.25 | 301153.88 | 12.63 |
